# An Adaptive Parallel Processing Strategy for Complex Event Processing Systems over Data Streams in Wireless Sensor Networks

**DOI:** 10.3390/s18113732

**Published:** 2018-11-02

**Authors:** Fuyuan Xiao, Masayoshi Aritsugi

**Affiliations:** 1 School of Computer and Information Science, Southwest University, No.2 Tiansheng Road, BeiBei District, Chongqing 400715, China; 2 Big Data Science and Technology, Division of Informatics and Energy, Faculty of Advanced Science and Technology, Kumamoto University, 2-39-1 Kurokami, Chuo-ku, Kumamoto 860-8555, Japan

**Keywords:** complex event processing, data streams, adaptive strategy, parallel processing, queue theory, probability theory

## Abstract

Efficient matching of incoming events of data streams to persistent queries is fundamental to event stream processing systems in wireless sensor networks. These applications require dealing with high volume and continuous data streams with fast processing time on distributed complex event processing (CEP) systems. Therefore, a well-managed parallel processing technique is needed for improving the performance of the system. However, the specific properties of pattern operators in the CEP systems increase the difficulties of the parallel processing problem. To address these issues, a parallelization model and an adaptive parallel processing strategy are proposed for the complex event processing by introducing a histogram and utilizing the probability and queue theory. The proposed strategy can estimate the optimal event splitting policy, which can suit the most recent workload conditions such that the selected policy has the least expected waiting time for further processing of the arriving events. The proposed strategy can keep the CEP system running fast under the variation of the time window sizes of operators and the input rates of streams. Finally, the utility of our work is demonstrated through the experiments on the StreamBase system.

## 1. Introduction

Recently, there has been an increasing interest in wireless sensor networks, which require continuously processing flowing data from geographically-distributed sources to achieve timely responses to complex queries, such as data stream processing (DSP) systems [1,2,3] and complex event processing (CEP) systems [4,5,6]. Additionally, the CEP systems focus on detecting the patterns of information that represent the higher level events, which are different with the DSP systems that focus on transforming the incoming flow of information [7]. Because the CEP system has many advantages, such as an expressive rule language and an efficient detection model of events, it has been highly concerned in academic circles and recently in industry [8,9,10,11,12,13,14]. In the CEP systems over data streams, events are processed in real time for all kinds of purposes, such as wireless sensor networks, financial tickers, traffic management, click-stream inspection and smart hospitals [15,16,17]. In these application domains, highly available event stream processing with a fast processing time is critical for handling the real-world events.

As far as we know, many kinds of parallel methods were devised to deal with massive distributed data streams for the DSP systems [18,19,20,21,22,23,24,25,26]. However, due to the differences between the DSP and CEP systems, most of the parallel methods that exclusively focus on aggregate queries or binary equi-joins in the DSP systems cannot be simply and directly used in the CEP systems that focus on multi-relational non-equi-joins on the time dimension, possibly with temporal ordering constraints, such as the sequence (SEQ) operator and conjunction (AND) operator [27,28]. Furthermore, the large volume and input rates of data streams are very common in big data applications [29,30]. The increased time window sizes of operators and input rates of streams may cause bottlenecks for the CEP system. Bottlenecks can slow down the CEP system. Even worse, they can result in the poor quality of query results, which has negative effects on decision-making.

To address these issues, we propose a parallelization model and an adaptive parallel processing strategy, called APPS by introducing histogram, probability theory and queue theory. The proposed APPS can estimate the optimal event splitting policy, which suits the most recent workload conditions such that the selected policy has the least expected waiting time for further processing of the coming events. Specifically, the CEP system based on the proposed parallelization model can split the input stream into parallel sub-streams to realize a scalable execution of continuous pattern query. APPS can keep the CEP system operating at high speed even under the variation of the time window sizes of the operators and input rates of the streams. The utility of our work is substantiated through the experiments on the StreamBase [12] system.

The rest of this paper is organized as follows. Section 2 discusses the related work in terms of the CEP systems. Section 3 briefly introduces the preliminaries of this paper. After that, a parallelization model and three event splitting policies are proposed in Section 4. Then, an adaptive parallel processing strategy is proposed to estimate and select the optimal event splitting policy to suit the workload conditions in Section 5. Section 6 demonstrates the utility of our proposal through the experiments on the StreamBase system. Finally, conclusions are given in Section 7.

## 2. Related Work

In CEP systems, the operators are demanded to be highly scalable under high event stream rates. It is well known that the traditional CEP systems are mostly centralized. For increasing the scalability of CEP systems, distributed parallelization processing is necessary. Until recently, studies were conducted and were mainly classified into two types: one focuses on the task parallelization of CEP systems; the other focuses on the data parallelization of CEP systems.

The task parallelization is also known as pipelining or intra-operator parallelization, where by deriving the states of operators and state transitions from the pattern query, the internal processing steps can be identified to be run in parallel. In particular, Suhothayan et al. [31] brought multi-threading and pipelining into CEP systems to make them process quickly. Wu et al. [32] presented a framework of parallelization for stateful operators over stream processing. Although these task parallelization methods are effective, they are not feasible for pattern matching. Later on, Balkesen et al. [33] devised a parallelization within a single partition of an event stream for scalable pattern matching. For the data parallelization, the main research works are as follows. Brito et al. [34] presented a system by combining the responsiveness of event stream processing systems with the scalability of the MapReduceprogramming model. Schneider et al. [35] introduced a compiler and run-time system, which could automatically extract data parallelism from streaming applications by partitioning the state through keys. De Matteis and Mencagli [36] presented parallel patterns for window-based stateful operators.

Through analyzing the existing works, it was found that the presented task parallelization and data parallelization methods were limited by the function of operators, especially for the pattern operators. Besides, some existing works that were based on key splitting were limited to the number of different key values. Most importantly, there is no adaptive parallel processing strategy for pattern operators in CEP systems. Therefore, in this paper, we focus on adaptive parallelization of pattern operators in CEP systems, which is a main contribution of this study. It is clear that this work contrasts withand is complementary to the previous works.

## 3. Preliminaries

### 3.1. Event Model

An event that represents an instance and is atomic is an occurrence of interest at a point in time. Basically, events can be classified into primitive events and composite events. A primitive event instance is a pre-defined single occurrence of interest that cannot be split into any small events. A composite event instance that occurs over an interval is created by composing primitive events.

**Definition** **1.** *A primitive event $e_{i}$ is typically modeled multi-dimensionally, denoted as $e_{i}$ = e($e_{i}.t$, ($e_{i}.st=e_{i}.et$), $<a_{1}$, …, $a_{m}>$), where, for simplicity, we use the subscript i attached to a primitive e to denote the timestamp i, $e_{i}.t$ is the event type that describes the essential features of $e_{i}$, $e_{i}.st$ is the start time-stamp of $e_{i}$, $e_{i}.et$ is the end time-stamp of $e_{i}$, $<a_{1}$, …, $a_{m}>$ are other attributes of $e_{i}$ and the number of attributes in e(·) denotes the dimensions of interest.*

**Definition** **2.** *Based on Definition 1, a composite event is denoted as e=e(e.t, (($e.st=\min_{1\leq i\leq n}e_{i}.st)<(e.et\;=\;\max_{1\leq i\leq n}e_{i}.et$)), $<a_{1}$, …, $a_{g}>$).*

### 3.2. Nested Pattern Query Language

We introduce the following nested complex event query language for specifying nested pattern queries:

PATTERN (event expression: composite event expressed by the nesting of SEQ and AND, which can have negative event type(s), and their combination operators)
WHERE (qualification: value constraint)
WITHIN (window: time constraint)

The composite event expression in the PATTERN clause specifies nested pattern queries, which support nests of SEQ and AND that can have negative event type(s), and their combination operators, as explained above. The negative event type in PATTERN means that the composite event is generated only when this event type has not occurred. Sub-expressions denote inner parts of a pattern query expression. The value constraint in the WHERE clause defines the context for the composite events by imposing predicates on event attributes. The time constraint in the WITHIN clause describes the time window during the time difference between the first and the last event instances, which is matched by a pattern query that falls within the window.

### 3.3. Pattern Operators and Their Formal Semantics

We define the operators that our method is targeting. Specifically, in this paper, we consider the pattern operators as presented in [37,38]. In the following, $E_{i}$ denotes an event type. More details were presented in [28].

**Definition** **3.** *An SEQ operator [37,38] specifies a particular order according to the start time-stamps in which the event must occur to match the pattern and thus form a composite event:*

$$\begin{array}{c} SEQ\;\left(E_{i},E_{j}\right)=\left\{<e_{i},e_{j}>|\left(e_{i}.st<e_{j}.st\right)\wedge \left(e_{i}.t=E_{i}\right)\wedge \left(e_{j}.t=E_{j}\right)\right\}. \end{array}$$

**Definition** **4.** *An AND operator [38] takes the event types as input, and events occur within a specified time window without a specified time order:*

$$\begin{array}{c} AND\left(E_{i},E_{j}\right)=\left\{<e_{i},e_{j}>|\left(e_{i}.t=E_{i}\right)\wedge \left(e_{j}.t=E_{j}\right)\right\}. \end{array}$$

## 4. System Model

### 4.1. Parallelization Model

**Figure 1:**
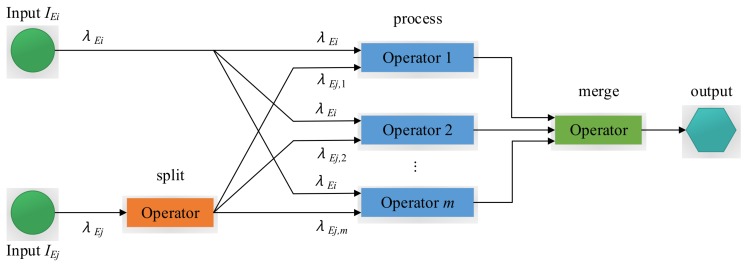
The parallelization model.

In this section, we propose a parallelization model that can be utilized for pattern operators, which is shown in Figure 1. We assume that each pattern operator is installed into a server (or host) here. Because of the specific property of pattern operators as described in Section 3, we cannot split both inputs $I_{E_{i}}$ and $I_{E_{j}}$ at the same time. Otherwise, this will omit detecting some events that may result in the wrong decision. Without loss of generality, an input stream can be randomly selected to be split, while the other one is replicated. Here, we assume splitting the input stream $I_{E_{j}}$ and replicate the other stream $I_{E_{i}}$. Specifically, once an event of $I_{E_{j}}$ arrives, the compute function of the pattern operator is initiated. In other words, the pattern operator creates a new window for every input tuple of $I_{E_{j}}$. Therefore, the input stream $I_{E_{j}}$ is split into parallel sub-streams that will be sent to back-end operators. The input rate of stream $I_{E_{j}}$ is equal to the sum of the input rates of sub-streams, i.e., $\lambda_{E_{j}}=\sum_{k=1}^{m}\lambda_{E_{j},k}$, where $\lambda_{E_{j},k}$ represents the input rate of a sub-stream to the back-end operator *k*. On the other hand, the replicate of input stream $I_{E_{i}}$ is directly sent to the back-end operators, each of which has input rate $\lambda_{E_{i}}$. We now provide details of the split−(process∗)−merge assembly, which facilitates the parallelization model of pattern operators.

As shown in Figure 1, the split−(process∗)−merge assembly replaces the solo pattern operator in the application data-flow. In the parallelized version of the application data-flow, $\lambda_{E_{j}}$ is split to the back-end process operators, and the output of the pattern operator is replaced by the output coming from the merge operator.

- Split: The split operator is to split an input stream into parallel sub-streams. The split operator outputs the incoming events to a number of back-end pattern operators by one of the event splitting policies from Section 4.2, where this selected event splitting policy is estimated by the adaptive parallel processing strategy that will be explained in Section 5.
- Process: The process operator performs the events from the output of the front-end operators. The multiple process operators with the same function can be executed in parallel.
- Merge: The merge operator consumes the output events from the process operators to generate the final output events. The merge operator by default simply forwards the output events to its output port.

### 4.2. Event Splitting Policies

In this section, the event splitting policies are given, which can be utilized for processing pattern operators in parallel.

• Round-robin (RR):

Events are assigned to the servers in a cyclical fashion, which means that the incoming events will be sent to the downstream servers with equal probability. This policy equalizes the expected number of events at each server.

• Join-the-shortest-queue (JSQ):

For the expected number of events, they are assigned to the downstream server with the shortest queue length for further processing. Here, the shortest queue means the queue with the fewest events.

• Least-loaded-server-first (LLSF):

For the expected number of events, it dynamically assigns them to the downstream server with the least load. The least loaded server is the server with the least used memory.

## 5. Adaptive Parallel Processing Strategy

**Figure 2:**
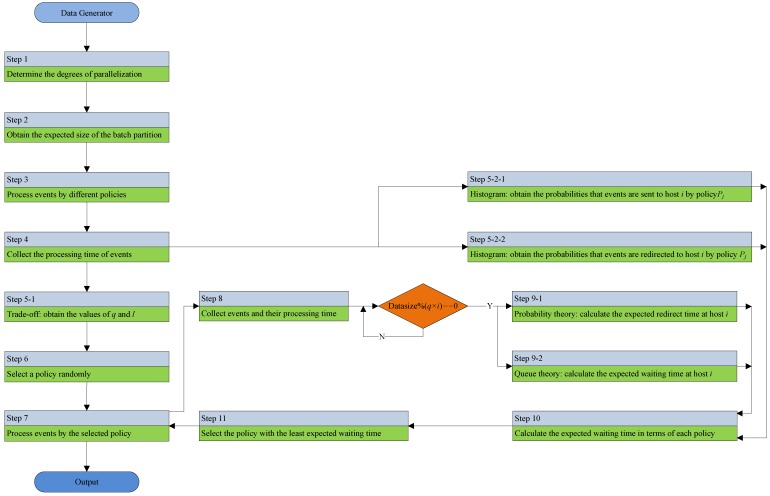
The flowchart of the adaptive parallel processing strategy.

In this section, an adaptive parallel processing strategy (APPS) is proposed to estimate and select the optimal event splitting policy, which can suit the most recent workload conditions such that the selected policy has the least expected waiting time for processing the coming events. Table 1 shows the key notations that are used in the remainder of this paper. Figure 2 describes the flowchart of the adaptive parallel processing strategy.

### 5.1. Degrees of Parallelization

The aim of this stage is to decide the degrees of parallelization for pattern operators in the CEP system to be used for processing data streams.

Let ρ be the expected server utilization, μ be the service rate, *m* be the number of servers and δ be the threshold of the expected server utilization that can be defined by the system administrator in advance according to the implication requirement. By applying queueing theory [39], ρ is given by:

(1) $$\begin{array}{cc} & \rho =\frac{\lambda }{m\mu },\\ s.t.\; & \rho \leq \delta ,\;0<\delta \leq 1. \end{array}$$

Based on Equation (1), we can obtain the degrees of parallelization for the pattern operator, i.e., the number of processing servers is as follows:

(2) $$m\geq \frac{\lambda }{\mu \delta },\;0<\delta \leq 1;m\in N.$$

### 5.2. Expected Size of the Batch Partition

For further parallel processing, the input stream $I_{E_{j}}$ needs to be divided into batch partitions. Because the number of events within each batch partition of segment $S_{\nu }$ of input stream $I_{E_{j}}$ should not exceed the threshold of the expected utilization of a single server, the number of events of a batch partition *i* should satisfy the following condition:

(3) $$\begin{array}{c} i=\mu \delta ,\;i\in N. \end{array}$$

### 5.3. Event Processing Time Collection

The aim of this stage is to collect the processing time of the events from the last event type matched by the pattern operator, which are used in the on-line estimation step to estimate various distributional properties of the processing time distribution.

For each new event arriving at the split operator, it records the arrival time of the event. These values are stored within the event. The arriving events of a segment of the input stream are then assigned to a back-end server by using the estimated policy $P_{j}$, where $P_{j}$ denotes the event splitting policy with the least expected waiting time to process the arriving events. Further details about how to select an appropriate event splitting policy on-line are discussed in Section 5.5. For each event that completes processing, its departure time will be stored at the assigned server. Next, the arrival time and departure time of the event will be sent to its back-end operator. Then, its corresponding processing time will be calculated by subtracting the departure time, which contributes to the last output event matched by the pattern operator that falls within the time window, from the arrival time recorded by the split operator.

### 5.4. Trade-Off between the Estimation Accuracy and the Processing Time

**Figure 3:**
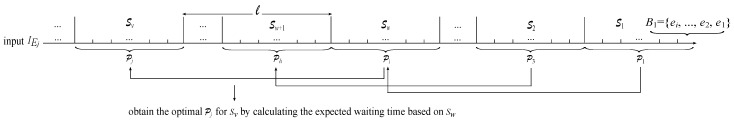
Example of obtaining an appropriate policy for processing the coming events.

Figure 3 depicts an example of obtaining an appropriate policy for processing the further coming events. $S_{\omega }$ denotes the $\omega^{\mathrm{th}}$ segment of input stream $I_{E_{j}}$. $B_{1}$ represents the first batch partition of the segment, which consists of events $\left\{e_{1},e_{2},\ldots ,e_{i}\right\}$. The policy $P_{j}$ under segment $S_{\nu }$ means these events in $S_{\nu }$ will be processed by using $P_{j}$ in which this estimated $P_{j}$ is selected based on the empirical data $S_{\omega }$. Therefore, we can notice that the time devoted to processing previous *ℓ* number of segments over *m* parallel servers should exceed the time devoted to estimating an appropriate policy $P_{j}$ for segment $S_{\nu }$. Otherwise, it introduces extra delay due to the waiting to obtain the optimal policy. In addition, to obtain the most accurate expected processing time for $S_{\nu }$, the mean squared error is considered.

Consequently, we treat the accuracy of estimation and the processing time of segments as an integrated constrained optimization problem. One objective ($O_{1}$) tries to maximize the accuracy of estimation, namely minimizing the mean squared error of estimation. On the other hand, the other objective ($O_{2}$) tries to maximize the processing time of segments to avoid introducing extra delay in selecting the optimal policy. Due to the conflicting nature of the different objectives, we obtain the solution by integrating them into one objective, and the optimization problem can thus be formulated as:

(4) $$\begin{array}{c} \min\frac{O_{1}}{O_{2}}. \end{array}$$

On the basis of the statement (4), the values of *q* and *ℓ* can be obtained and be used in the further on-line event splitting policy selection procedure in Section 5.5.

Objective $O_{1}$: mean squared error of estimation constraint.

In this paper, a general expression is derived for the expected waiting time by applying queueing theory [40], denoted as f(E[W]). Let ${\hat{f}}_{S_{\omega }}\left(E\left[W\right]\right)$ be the expected processing time of the events of segment $S_{\nu }$ in terms of empirical data $S_{\omega }$. Then, ${\hat{f}}_{S_{\omega }}\left(E\left[W\right]\right)$ is compared with $f_{S_{\left(\nu -1\right)}}\left(E\left[W\right]\right)$ by the following mean squared error (MSE):

(5) MSE=1τq−ℓ−1∑ω=ν−1−ℓ,ν=ℓ+2τq(f^Sω(E[W])−fS(ν−1)(E[W]))2,withf(E[W])=ρ2(m+1)−1μm(1−ρ)(Ca2+Cs22),1≤q,ℓ≤τ;1≤ω≤τq,s.t.MSE<β,

in which *q* is the number of batch partitions of segment $S_{\nu }$ and *ℓ* is the difference value subtracting (ν−1) of $S_{\nu -1}$ from ω of $S_{\omega }$, which is devoted to estimating policy $P_{j}$ for segment $S_{\nu }$. $C_{a}^{2}$ represents the squared coefficient of variation of inter-arrival times, and $C_{s}^{2}$ represents the squared coefficient of variation of service times where they can be obtained by testing. $f_{S_{\left(\nu -1\right)}}\left(E\left[W\right]\right)$ is the true expected processing time of the events of segment $S_{\nu }$ in terms of empirical data $S_{\left(\nu -1\right)}$, because it is the nearest empirical data of $S_{\nu }$ to obtain the most accurate expected processing time. β denotes the threshold of the mean squared error of estimation that can be defined by the system administrator in advance according to the implication requirement. 

Objective $O_{2}$: processing time constraint. 

Let ${\bar{T}}_{ps}^{i}$ be the time devoted to processing *i* number of events, ${\bar{T}}_{rd}^{i+1}$ be the time devoted to re-directing the ${\left(i+1\right)}^{\mathrm{th}}$ event and ${\bar{T}}_{es}^{i}$ be the estimate time for *i* number of events. The objectives $O_{2}$ should satisfy the following condition:

(6) $$\begin{array}{cc} & {\bar{T}}_{ps}^{S}=q{\bar{T}}_{ps}^{i}+\left(q-1\right){\bar{T}}_{rd}^{i+1},\\ s.t.\; & \ell \frac{{\bar{T}}_{ps}^{S}}{m}>T_{es}^{P_{j}},\\ with\; & T_{es}^{P_{j}}=q{\bar{T}}_{es}^{i},\\ & 1\leq q,\ell \leq \tau . \end{array}$$

The values of ${\bar{T}}_{ps}^{i}$, ${\bar{T}}_{rd}^{i+1}$ and ${\bar{T}}_{es}^{i}$ can be obtained via testing. If the number of events within one batch partition of $S_{\nu }$ is large enough, while the time for re-directing each batch partition is quite smaller than the time for processing each batch partition, we can omit ${\bar{T}}_{rd}^{i+1}$ in Equation (6).

### 5.5. On-Line Selection of Event Splitting Policies

This stage is pretty critical in the proposed adaptive parallel processing strategy, which can estimate and decide the appropriate policy on-line. In order to calculate the expected waiting time for the policies, we first leverage the histogram to obtain the probabilities that the events are sent to host *i* by policy $P_{j}$, denoted as $P_{j}^{H_{i}}$, and the probabilities that the events are redirected to host *i* by policy $P_{j}$, denoted as $P_{j}^{R_{i}}$.

Next, we introduce queue theory [41] to get the expected waiting time for the events at host *i*, which is formulated as:

(7) $$\begin{array}{c} E\left[W_{i}^{H}\right]=\frac{1}{\mu_{i}}\left(\frac{\rho_{i}}{1-\rho_{i}}\right)\left(\frac{C_{ia}^{2}+C_{is}^{2}}{2}\right), \end{array}$$

where $\rho_{i}$ denotes the expected server utilization at host *i*, $\mu_{i}$ represents the number of events served per unit time at host *i*, $C_{ia}^{2}$ represents the squared coefficient of variation of inter-arrival times at host *i* and $C_{is}^{2}$ represents the squared coefficient of variation of service times at host *i* where they can be obtained on-line.

Additionally, we utilize probability theory to calculate the expected redirect time for the events at host *i*, which is formulated as:

(8) $$\begin{array}{c} E\left[W_{i}^{R}\right]=\sum_{r=1}^{k}x_{r}f\left(x_{r}\right). \end{array}$$

Based on the probabilities that events are sent and redirected to different hosts, the expected waiting time for the events at different hosts and the expected redirect time at different hosts, we then calculate the expected waiting time for all the policies in the list of APPS. APPS derives a general expression for the expected waiting time for policy $P_{j}$, denoted as $E[W_{P_{j}}]$, by applying probability theory to select the event splitting policy with the least expected waiting time, which can be formulated as:

(9) $$\begin{array}{c} E\left[W_{P_{j}}\right]=\sum_{i=1}^{h}\left(P_{j}^{H_{i}}E\left[W_{i}^{H}\right]+P_{j}^{R_{i}}E\left[W_{i}^{R}\right]\right). \end{array}$$

## 6. Experimental Evaluation

Based on the parallelization model in Figure 1, we implemented the experiments on the StreamBase [12] system for query $q_{1}$.

$$\begin{array}{cc} q_{1}:\; & \mathrm{PATTERN}\;SEQ\;(E_{1},E_{2})\\ & \mathrm{WHERE}\;[\mathrm{Id}]\\ & \mathrm{WITHIN}\;1\;s \end{array}$$

Since the proposed method both contrasts withand is complementary to the existing methods, APPS is compared with the RR, JSQ and LLSF methods to prove the utility and effectiveness of the proposed method. We ran the experiments on the machines, each of which has an AMD Opteron(tm) Processor 6376 and 4.00 GB main memory. Streams used in the experiments were generated synthetically. Specifically, each stream was set with three attributes, including the event id, time-stamp and event type, in which the incoming events of streams were uniformly distributed. We define the processing time as the difference between the departure time, which contributes to the last output event matched by the pattern operator that falls within the time window, and the arrival time recorded by the split operator. For the simplicity of the experiments, we provided four machines for APPS, RR, JSQ and LLSF: one machine that created input data and split the input stream into back-end machines, another two machines that were equipped with SEQ operators with the same functions to process the input streams in parallel and another machine that received data and output the throughput. Then, we compared the performance of these methods under different parameter settings in terms of input rate and time window size.

### 6.1. Comparing the Processing Time of the Methods

**Figure 4:**
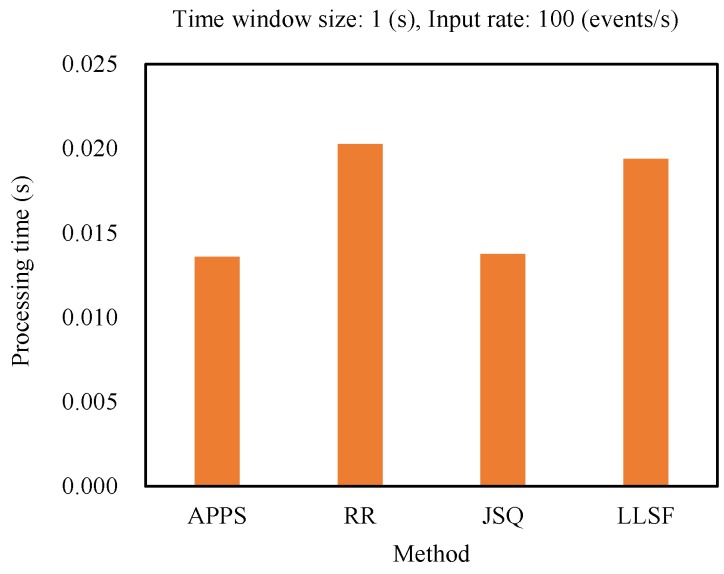
Comparing the processing time of the methods (APPS: An adaptive parallel processing strategy, RR: Round-robin, JSQ: Join-the-shortest-queue, LLSF: Least-loaded-server-first).

In this experiment, the input rates were set as 100 events/s, and time window sizes were set as 1 s. From the experimental result as shown in Figure 4, it was obvious that APPS and JSQ had lower processing time compared with the RR and LLSF methods. Because APPS could estimate and select the optimal event splitting policy for further processing of the coming events, it had almost the same processing time as the JSQ method.

### 6.2. Varying the Time Window Sizes of Operators

**Figure 5:**
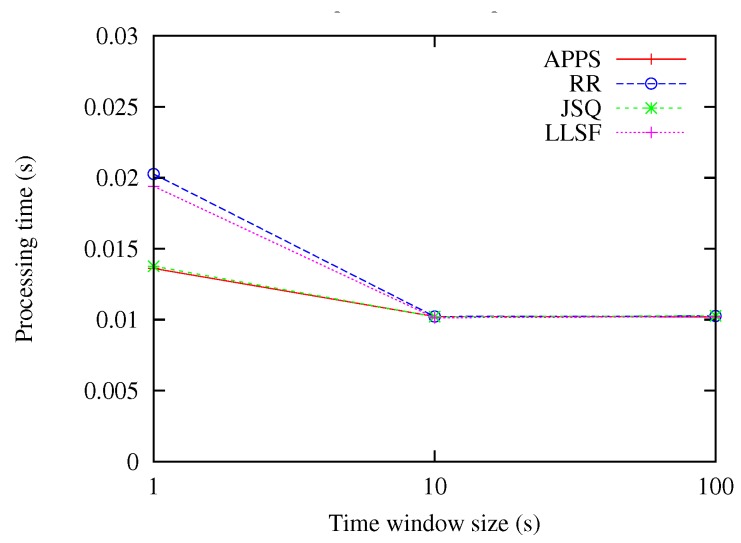
Comparing the methods under the variation of time window sizes.

In this experiment, the input rates were set as 100 events/s, while the time window sizes were varying from 1 up to 10, and 100 s. From the experimental result as shown in Figure 5, we can notice that APPS had almost the same processing time as the JSQ method, and both outperformed the RR and LLSF methods, especially when the time windows sizes were set as 1 s; whereas, as the time windows sizes increased from 10 up to 100 s, the APPS, RR, JSQ and LLSF methods almost had the same performance. The reason is that as the time windows sizes increased from 10 up to 100 s, it reached the limitation of the processing capacity of the machines.

### 6.3. Varying the Input Rates of Streams

**Figure 6:**
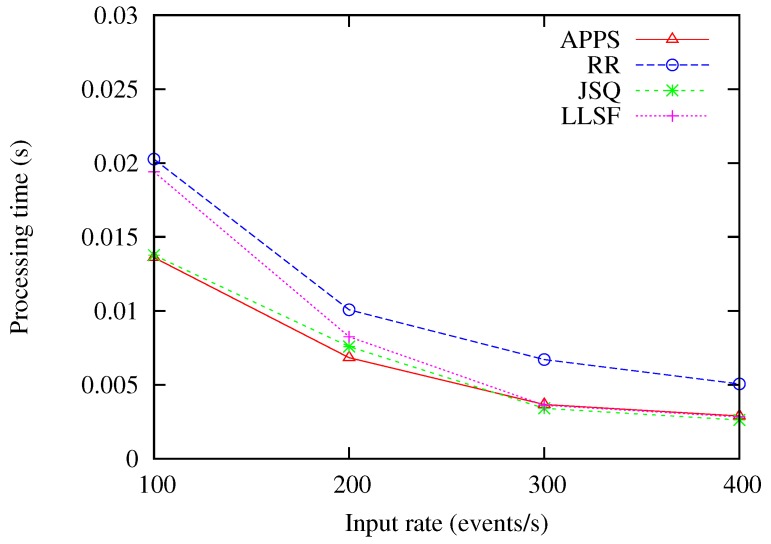
Comparing the methods under the variation of input rates.

In this experiment, the time window sizes were set as 1 s, while the input rates were varying from 100 up to 200, 300 and 400 events/s. From the experimental result as shown in Figure 6, we can obviously see that the performance of APPS was significantly better than the performance of the RR, JSQ and LLSF methods. Because APPS, which suits the most recent workload conditions, estimated and selected the optimal event splitting policy for further processing of the coming events, it could handle the input rate variation environment. On the other hand, as the input rates increased from 100 up to 200, 300 and 400 events/s, the JSQ and LLSF methods had a lower processing time than the RR method, because JSQ assigned the events to the back-end server with the shortest queue length, while LLSF assigned the events to the back-end server with the least load for further processing of the coming events.

## 7. Conclusions

In this paper, we started off with identifying the general problems of adaptive parallel processing with respect to pattern operators in CEP systems. We proposed a new adaptive parallel processing strategy to estimate the optimal event splitting policy, which can suit the most recent workload conditions such that the selected policy had the least expected waiting time for further processing the coming events. Moreover, because the proposed method was a hybrid method of intra-operator parallelization and data parallelization, our proposal was not limited to the number of different key values. The proposed strategy kept the CEP system running with fast processing under the variation of the time window sizes of operators and input rates of streams. The utility of our work was demonstrated through the experiments on the StreamBase system.

The proposed adaptive parallel processing strategy considered only the SEQ and AND operator-based pattern query in this study. Thereby, in the future work, more complex operators will be further investigated for the pattern queries, such as the nested SEQ and AND operators, which may have negative event types, and combinations of them. In addition, we intend to achieve a pilot implementation of the framework, where more complicated experimental environment and performance analysis will be taken into account in the future work, including the Poisson distribution, the exponential distribution of incoming events, etc. [42,43]. Another interesting future work is about how to detect complex events over probabilistic event streams adaptively. In view of the efficiency in handling uncertainty, some useful extended methods, like fuzzy theory, evidence theory, probability and the entropy-based method [44,45,46,47], will be considered in CEP systems in the future work.

## Figures and Tables

**Table 1:** Notation.

Notation	Meaning
$P_{j}$	event splitting policy *j*
ρ	the expected server utilization
δ	threshold of the expected server utilization
*m*	degree of parallelization of servers
μ	number of events served per unit time
$\lambda_{E_{j}}$	input rate of input stream $I_{E_{j}}$
$S_{\nu }$	the $\nu^{\mathrm{th}}$ segment of input stream $I_{E_{j}}$
$B_{g}$	the $g^{\mathrm{th}}$ batch partition of a segment
*i*	number of events of a batch partition
*q*	number of batch partitions of a segment
${\bar{T}}_{ps}^{i}$	average time devoted to processing *i* number of events
${\bar{T}}_{rd}^{i+1}$	average time devoted to re-directing the ${\left(i+1\right)}^{\mathrm{th}}$ event
	among servers
${\bar{T}}_{ps}^{S}$	average time devoted to processing segments
${\bar{T}}_{es}^{i}$	average estimation time devoted for *i* number of events
$T_{es}^{P_{j}}$	estimation time devoted to obtaining optimal $P_{j}$ for $S_{\nu }$
$E[W_{i}^{R}]$	expected redirect time for the events at host *i*
$E[W_{i}^{H}]$	expected waiting time for the events at host *i*
$E[W_{P_{j}}]$	expected waiting time for policy $P_{j}$
